# *Ltbp1L* is focally induced in embryonic mammary mesenchyme, demarcates the ductal luminal lineage and is upregulated during involution

**DOI:** 10.1186/bcr3578

**Published:** 2013-11-21

**Authors:** Anupama Chandramouli, Julia Simundza, Alicia Pinderhughes, Minoti Hiremath, Gustavo Droguett, David Frendewey, Pamela Cowin

**Affiliations:** 1 Department of Cell Biology, New York University School of Medicine, New York, NY, USA; 2 The Ronald O Perelman Department of Dermatology, New York University School of Medicine, 550 First Ave, New York, NY 10016, USA; 3 Regeneron Pharmaceuticals, 777 Old Saw Mill River Rd, Tarrytown, NY 10591, USA

## Abstract

**Introduction:**

Latent TGFβ binding proteins (LTBPs) govern TGFβ presentation and activation and are important for elastogenesis. Although TGFβ is well-known as a tumor suppressor and metastasis promoter, and LTBP1 is elevated in two distinct breast cancer metastasis signatures, LTBPs have not been studied in the normal mammary gland.

**Methods:**

To address this we have examined *Ltbp1* promoter activity throughout mammary development using an Ltbp1L-LacZ reporter as well as expression of both Ltbp1L and 1S mRNA and protein by qRT-PCR, immunofluorescence and flow cytometry.

**Results:**

Our data show that *Ltbp1L* is transcribed coincident with lumen formation, providing a rare marker distinguishing ductal from alveolar luminal lineages. *Ltbp1L* and *Ltbp1S* are silent during lactation but robustly induced during involution, peaking at the stage when the remodeling process becomes irreversible. *Ltbp1L* is also induced within the embryonic mammary mesenchyme and maintained within nipple smooth muscle cells and myofibroblasts. Ltbp1 protein exclusively ensheaths ducts and side branches.

**Conclusions:**

These data show *Ltbp1* is transcriptionally regulated in a dynamic manner that is likely to impose significant spatial restriction on TGFβ bioavailability during mammary development. We hypothesize that Ltbp1 functions in a mechanosensory capacity to establish and maintain ductal luminal cell fate, support and detect ductal distension, trigger irreversible involution, and facilitate nipple sphincter function.

## Introduction

Latent transforming growth factor β (TGFβ) binding proteins (LTBPs) are regulators of elastogenesis and TGFβ [1]. Their critical role in tissue development, homeostasis and resilience is demonstrated by the fact that *LTBP* loss-of-function mutations underpin a growing list of human genetic syndromes [2-4]. Gain of *LTBP* gene expression also has pathological consequences: *LTBP1* is upregulated in two breast cancer metastasis signatures and is one of only six genes found in common to both [5,6].

*Ltbp* genes encode a family of secreted proteins, Ltbp1-4, that show extensive sequence homology to fibrillins, which polymerize to form microfibrils and coat elastic fibers [1,7]. Ltbp proteins are initially deposited onto fibronectin and later transferred to microfibrils by interaction with fibrillins [8]. Their importance for the structural integrity and tensile function of the extracellular matrix (ECM) is illustrated by the pathologies seen in *Ltbp4S*-null mice resulting from defective elastic-fiber formation in the intestine, lung and pulmonary artery and in humans with Urban-Rifkin-Davis syndrome [4,9,10].

In addition to their contribution to ECM structure, Ltbp1, Ltbp3 and to a lesser extent Ltbp4 govern the spatial patterning and activation of TGFβ. TGFβs are secreted in a latent form, encapsulated by their cleaved latency-associated propeptide (LAP), and deposited within the ECM for subsequent activation. Ltbps post-translationally regulate TGFβ in three ways. First, they chaperone the association of TGFβ with LAP and through preferential binding affinities control which of three TGFβ isoforms emerge from the cell [11]. Second, Ltbps incorporate latent TGFβ within the ECM thereby determining where TGFβ is presented to its receptors [12]. Third, Ltbps provide a key link between the ECM and the cell surface that is essential for stretch activation of TGFβ [13-15]. Both integrins and Ltbp bind to LAP. Thus, when Ltbp1 is anchored in a stiff ECM and stress fibers exert tension on integrins, conformational changes occur in LAP that lead to release of the active TGFβ [13,14,16]. One major response to TGFβ signaling is synthesis of new matrix proteins [17]. Thus, Ltbps create a mechanosensory system that generates a highly localized feedback response to cell traction or tension within the microenvironment [1,18].

Mouse mutants have illuminated the roles of Ltbps in tissue homeostasis and their involvement in human pathology. *Ltbp1* hypomorphs show facial dysmorphia [19] and *Ltbp1L* loss leads to embryonic lethality due to heart malformation [20], *Ltbp2* loss-of-function mutations cause glaucoma in humans and lens defects in mice [21], *Ltbp3* loss-of-function mutation results in severe bone malformation [3,22,23] and *Ltbp4S*-null mice show multiple organ defects [4,9,10]. In some mutants the prevailing pathology reflects compromised elastogenesis [10,24]. In others the phenotype can be ameliorated by concurrent deletion or pharmacological antagonism of TGFβ, supporting the central role of Ltbps in TGFβ biology and pathology [10].

Three TGFβ isoforms are differentially expressed and exert multiple effects during mammary development [25]. Loss- and gain-of-function studies have shown that TGFβ signaling restrains pubertal ductal extension and side branching by stimulating Wnt5a expression [26-31]. TGFβ1 influences stem cell regenerative potency and cell-fate determination and has been proposed to suppress precocious alveologenesis in the adult gland prior to pregnancy [27,32-36]. Weaning massively induces TGFβ3 expression, and this surge is essential for the demise of the differentiated glandular epithelium and remodeling events during mammary involution [37,38]. TGFβ1 has also been the object of intense investigation due to its pathological relevance for breast cancer [39,40] where it acts as a tumor suppressor in premalignant lesions and at later stages promotes metastasis through induction of epithelial-to-mesenchymal transition (EMT).

Knowledge of Ltbp's temporal and spatial expression pattern is central to understanding TGFβ signaling both in the physiological setting of the normal mammary gland and in breast cancer. Yet to date there have been no studies on Ltbp within the normal mammary gland. Here we show that *Ltbp1* is induced in a highly specific temporal and spatial pattern throughout mammary development, supporting the concept that dynamic transcriptional regulation of *Ltbp1* provides a mechanism to impose considerable restriction on TGFβ bioavailability. *Ltbp1L* is upregulated early during embryonic mammary mesenchyme specification and is sustained in smooth muscles of the nipple sphincter. Within the mammary gland, *Ltbp1L* is induced exclusively in the ductal luminal epithelium but is silent in alveoli and therefore provides a rare biomarker distinguishing ductal from alveolar luminal lineages. Ltbp1 protein is deposited around basal cells of all ducts and side branches, and lies in close proximity to elastic fibers that exclusively encase the permanent ductal system. *Ltbp1* is prominently upregulated during involution, with kinetics similar to that reported for TGFβ3, suggesting important functions in gland remodeling.

## Methods

### Mice

**Figure 1:**
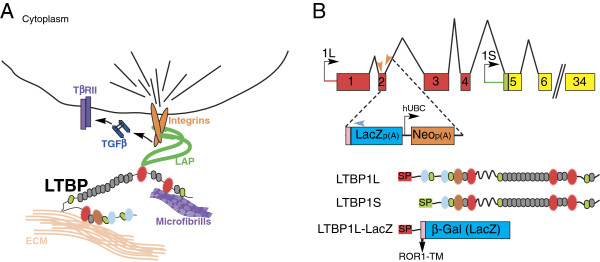
**Latent TGFβ binding protein (Ltbp) function and reporter construct. (A)** Ltbp1 sequesters TGFβ ligand encased by its latency associated propeptide (LAP) within the extracellular matrix (ECM). Integrins also interact with LAP. Cytoskeletal tension on integrins stretches LAP and releases TGFβ locally to activate the TGFβ receptor II (TβRII). **(B)** Two Ltbp1 isoforms (1L and 1S) are transcribed from distinct promoters (black arrows, top). The first four exons (red boxes) encode sequence unique to Ltbp1L. The promoter, transcription start site and unique signal peptide sequence for Ltbp1S (green line and box) lie within the 4th intron of *Ltbp1L*. Black lines indicate introns. The targeted deletion of *Ltbp1L* replaces codon 165 in exon 2 through 7.8 kb of intron 2 with a cassette comprising the ROR1 transmembrane domain (pink box), a modified *lacZ* gene and polyadenylation signal (blue box) as well as a floxed neomycin gene (orange box) driven by the human ubiquitin binding complex promoter (hUBC) that was removed by cre recombination in embryonic stem cells. Orange and blue arrowheads indicate primers used for genotyping. 1L and 1S isoforms contain a signal peptide (SP), 4-Cys (blue), 8-Cys (red) and epidermal growth factor (EGF)-like repeats (gray and green), and unique 8-cys/EGF hybrid domains (brown). The Ltbp1L-LacZ reporter comprises the signal peptide and the first 165 amino acids of Ltbp1L protein followed by the ROR1 transmembrane domain fused in-frame with β-galactosidase and lacks all functional LTBP1 domains.

*Ltbp1L*^*lz/+*^ mice, were generated by Regeneron Pharmaceuticals, Inc., Tarrytown, NY. VelociGene methods [41] were used to recombineer a bacterial artificial chromosome (BAC) clone, such that a region extending from the 165th codon of murine *Ltbp1L* in exon 2 through the remainder of exon 2 and 7.8 kb into the downstream intron, was replaced by homologous recombination with an expression cassette comprising the transmembrane domain of ROR1 fused in-frame with the upstream coding sequence of *Ltbp1L*, followed by a stop-transfer sequence, a modified β-galactosidase coding sequence (*lacZ*), a polyadenylation signal and an antibiotic selection cassette flanked by *loxP* sites [42] (see Figure 1). The modified BAC, was linearized, producing 5′ and 3′ homology arms of approximately 150 kb and 30 kb flanking the deletion, and electroporated into SvEv129/C57Bl6/F1-derived hybrid embryonic stem (ES) cells. Targeting of ES cells and the germline transmission were confirmed by a quantitative reverse transcriptase PCR (qRT-PCR) assay that scored for the loss of one of the native *Ltbp1L* alleles [41]. The *neo*^*R*^ cassette was removed by crossing with mice expressing Cre recombinase in the germ-cell lineage and the knock-out was confirmed by northern and western analysis [20]. Ltbp1L^lz/+^ mice on a mixed C57Bl6/129 background were rederived into the Skirball animal facility and crossed onto an FVBN strain background by breeding through nine generations. All animal protocols were approved by the Institutional Animal Care and Use Committee (IACUC) of New York University School of Medicine.

Carmine staining of mammary whole mounts revealed no differences between *Ltbp1L*^*lz/+*^ mice and wild-type littermates in ductal elongation, branching, alveolar development or involution. Pups from both genotypes faired equally well in terms of weight gain (data not shown). We concluded that *Ltbp1L*^*lz/+*^ mice show no evidence of haploinsufficiency and justified their use to study the regulated expression of *Ltbp1L* during mammary development. Staging of pregnancy and embryos were performed by daily checking of vaginal plugs, with noon of the day of the plug considered day 0.5. Embryonic stages were confirmed by determining the degree of limb development as indicated in Theiler’s classification of mouse development (*The Atlas of Mouse Development*, MH Kaufman).

### Genotyping

Mice and embryos were screened by 5-bromo-4-chloro-3-indolyl-β-D-galactoside (X-Gal) staining of tails and confirmed by PCR analysis. Genomic DNA was prepared from 0.5 cm of tail by digesting overnight in 0.5 ml digestion buffer (50 mM Tris–HCl pH 7.4, 100 mM ethylenediaminetetraacetic acid (EDTA), 100 mM NaCl, 0.5% SDS, 200 μg/ml proteinase K). Then 150 μl of 5 M NaCl was added and the digest was agitated for 15 minutes on a rotator: 500 μl of supernatant was collected after centrifugation at 14,000 G for 15 minutes, and subjected to two rounds of ethanol precipitation. The final pellet was resuspended in 200 μl TE (10 mM Tris–HCl pH 7.4, 1 mM EDTA) and 1 μl was added to a 20-μl PCR. Thirty cycles of PCR (94°C, 58°C and 72°C for 1 minute each) were carried out. The wild-type *Ltbp1L* allele was detected by amplification of a 430-bp band using forward 5′-CTTAGTTCCTCCATCCTTCC-3′ and reverse 5′-CAGACTTCACCTTCCCAGGG-3′ primers. The Ltbp1L^lz/+^ knock-in allele was detected in a separate reaction using the forward primer listed above and a reverse primer 5-GTCTGTCCTAGCTTCCTCACTG-3′ (see Figure 1B arrowheads) to amplify a 440-bp product. The gender of embryos was determined by amplification of the *Sry* gene on the Y chromosome (forward primer: 5′-GAGAGCATGGAGGGCCAT-3′ and reverse primer: 5′-CCACTCCTCTGTGACACT-3′). Amplification products were resolved by electrophoresis on 2% agarose gels run for 30 minutes in TAE electrophoresis buffer (40 mM Tris-acetate, 1 mM EDTA).

### X-Gal staining of embryos and mammary gland whole mounts

Embryonic day (E) 10.0 to E15.5 embryos were dissected and fixed in 4% paraformaldehyde (PFA) (Sigma Aldrich, St Louis, MO, USA) prepared in PBS for 20 to 50 minutes depending on the stage. Skin with attached mammary fat pads was removed from E16.5 to E18.5 embryos and stretched carefully on cardboard, and mammary glands from adult mice were dissected and flattened onto glass slides then fixed in 4% PFA for 30 minutes. Following fixation, samples were washed 4 × 15 minutes with rinse buffer (2 mM MgCl_2_, 0.1% sodium deoxycholate, 0.2% NP40 prepared in PBS) and stained in X-Gal staining solution (5 mM potassium ferricyanide, 5 mM potassium ferrocyanide, 1 mg/ml 5-bromo-4-chloro-3-indolyl-b-D-galactopyranoside (X-Gal, Denville Scientific, South Plainfield, NJ, USA) prepared in rinse buffer) at room temperature for 2 to 3 h. After staining, samples were rinsed twice in PBS and post-fixed in 4% PFA overnight at 4°C, dehydrated through an ethanol gradient (2 × 10 minutes in 70%, 95%, and 100% ethanol), then placed in Carnoys’s fixative (60% ethanol, 30% chloroform, 10% glacial acetic acid) followed by Citrisolv reagent (Fisher Scientific, Pittsburgh, PA, USA) to clear the fat.

### Whole-mount carmine staining

X-Gal stained mammary glands were rehydrated in a reverse-graded series of ethanol washed in water and then stained for 1 h in carmine solution diluted 1:5 in water. Carmine was prepared by boiling 1 g carmine alum and 2.5 g aluminium potassium sulphate in 500 ml of water for 20 minutes followed by filtration. The glands were dehydrated in a graded ethanol series, cleared in Carnoy’s solution, placed in Citrisolv for 30 minutes, and mounted in Cytoseal (VWR, Radnor, PA, USA). Glands were then viewed using a Zeiss Axiovert (Oberkochen, FRG) brightfield microscope.

### Histology and immunodetection

E10.5-stage embryos were embedded in 10% gelatin, sectioned at 70 μm with a vibratome, and mounted with Fluoromount G (Southern Biotech, Birmingham, AL, USA). Older embryos and mammary glands were processed for X-Gal staining and fixation as described above. Isopropanol was substituted for xylene to prevent diffusion of the X-Gal stain during processing and tissues were embedded in paraffin and sectioned. Sections (4 μm) were placed on Superfrost Plus slides, baked 1 h at 60°C and deparaffinized for 5 minutes in Citrisolv for X-Gal-stained tissues. Tissues were then rehydrated through a reverse gradient of ethanol solutions. For histology, sections were stained with 0.1% solution of Nuclear Fast Red (NFR) (Polyscientific, Bayshore, NY, USA) for 1 minute. Tissues were then dehydrated and dipped in xylene (or Citrisolv in the case of X-Gal-stained tissues) before being mounted in Cytoseal (VWR). For immunohistochemistry (IHC), citric acid antigen retrieval was performed by submerging the slide containing deparaffinized 4-μm sections in 10 mM sodium citrate solution (pH 6.0) and boiling in a microwave at 90% power for 30 minutes, followed by quenching of endogenous peroxidase using 3% hydrogen peroxide. Primary mouse antibodies against smooth-muscle actin (SMA) 1 (1:500, DAKO, Carpinteria, CA, USA), estrogen receptor (1:500, DAKO), p63 (1:1,000 LabVision, Kalamazoo, MI, USA), and rabbit antibodies against Cytokeratin 14 (1:8,000, Covance, Princeton, NJ, USA), Lef-1 (1:100 Cell Signaling, Danvers, MA, USA), androgen receptor (1:500, Santa Cruz Biotechnologies, Santa Cruz, CA, USA) and guinea pig antibodies against Vimentin (1:1,000, Progen) were added overnight at 4°C. For IHC, biotin-labeled secondary antibodies (1:1,000) and streptavidin-horseradish peroxidase (HRP) (1:200, Vector Laboratories, Burlingame, CA, USA) were added for 30 minutes each, and colorimetrically detected with diaminobenzidine (Vector Labs). Frozen 5-μm sections were stained with rabbit antibodies against LTBP (Ab39 [43], 1:200, a gift from Dr Carl-Henrik Heldin, Uppsala University, Sweden, and rL1C [44], 1:100, a gift from Dr Lynn Sakai, Portland Shriners Research Center, Portland, OR, USA), tropoelastin (1:500, Elastin Products Company, Inc., Owensville, MO, USA), and mouse anti-SMA, described above, were detected by Cy3-labeled donkey anti-rabbit (Fisher Scientific) and Alexafluor-488-labeled donkey anti-mouse secondary antibodies (Life Technologies Inc, Carlsbad, CA, USA). Bioreagent (4′,6-diamidino-2-phenylindole dihydrochloride (DAPI) from Sigma Aldrich) was used for immunofluorescent localization of nuclei in confocal images. Elastic fibers were also detected by staining with Wiegert’s resorcin-fuchsin for 1 minute [45].

### Mammary epithelial cell preparation and flow cytometry

The third, fourth and fifth mammary glands from 8- to 16-week-old virgins were dissected, inguinal lymph nodes were discarded, and the mammary glands were minced between two scalpels into a fine paste. The tissue was dissociated for 6 h at 37°C in collagenase/hyaluronidase solution (catalog number 07912, Stem Cell Technologies Inc., Vancouver, BC, Canada), and further dissociated with 0.25% Trypsin-EDTA and 10 mg/ml dispase (catalog number 07913, Stem Cell Technologies) with 1 mg/ml DNase, before filtering through a 40-μm mesh. Endothelial and hematopoietic lineages were depleted using antibodies to TER119, CD45, CD140a, and CD31 (1:100, Becton Dickenson (BD), Franklin Lakes, NJ), with three separations on an EasySep magnet. Primary antibodies CD49f-PerCP-Cy5.5 (1:200, BD), CD24-PE (1:400, BD), CD29-Pacific Blue (1:200, Biolegend, San Diego, CA, USA), CD61-APC (1:200, CalTag MedSystems, Buckingham, UK), stem cell antigen 1 (Sca1)-phycoerythrin (PE) (1:400, BD) were added for 30 minutes at 4°C. Fluorescein Di-β-D-Galactopyranoside (FDG-gal) loading was performed after primary antibody staining, according to the manufacturer’s instructions (FluoReporter Kit, Life Technologies, Green Island, NY, USA). Flow cytometry was performed on a BD LSRII or BD FacsCalibur, and analyzed using FlowJo v8.7.

### RNA isolation and qRT-PCR analysis

The fourth and fifth pair of mammary glands were harvested from wild-type mice at different stages of postnatal mammary development, dissected and snap-frozen in liquid nitrogen. A block of tissue approximately 0.5 × 0.5 × 0.5 cm was homogenized for 5 minutes in 1 ml of TRI-Reagent (Life Technologies) using a hand-held tissue homogenizer (Kinematica, Lucerne, Switzerland), then mixed with 200 μl of chloroform and centrifuged at 14,000 G for 15 minutes to eliminate protein debris. The upper aqueous phase was mixed with an equal volume of 70% ethanol and passed through a Qiagen RNeasy mini spin column by a brief 15 sec centrifugation at 8,000 G at room temperature. Total RNA bound to the column filters was washed in 350 μl of ethanol-containing buffer (RW1 buffer; Qiagen, Valencia, CA, USA) to remove contaminants and incubated in 10 μl of RNase-free DNase I enzyme (273 Kunitz units; Qiagen) for 15 minutes at room temperature to ensure digestion of any residual genomic DNA fragments. The columns were washed according to the manufacturer’s instructions in ethanol-containing buffers (RW1 and RPE buffers; Qiagen). Total RNA was eluted in 50 μl of RNase-free water, and its concentration was analyzed by Nanodrop measurement. Reverse transcription was performed using 2 μl of RNA (10 ng/μl) from tissue using the QuantiTect Probe RT-PCR Kit (Qiagen; catalog number 204443). Real-time analysis was performed using the Taqman Gene Expression Assay (Applied Biosystems by Life Technologies; catalog number 4331182) for mouse *Ltbp1* (Mm00498255_m1), *Ltbp1L* (Mm01226402_m1 spanning exons 1 and 2), and *Ltbp1S* (custom assay with forward primer: 5′-TTCCAAGGCAAGTTCATGGATA-3′, within intron 4; reverse primer: 5′-AGGAGTAGAGGCAGACAGAGAAAGA-3′, within the fifth exon of *Ltbp1* genomic sequence and MGB probe: 5′-6FAM-TAAGCTGATGTGTTTGTTG-3′-MGBNFQ) and mouse β2-microglobulin (Mm00437762_m1). Real-time analysis was performed in the Applied Biosystems ViiA™ 7. Total Ltbp1, Ltbp1L and 1S mRNA levels were normalized to those of mouse β2-microglobulin and plotted as levels relative to tissue from 12-week-old virgins.

## Results

### Ltbp1L-LacZ expression underlies a route for axillary cell migration and is an early marker of the mammary mesenchyme

**Figure 2:**
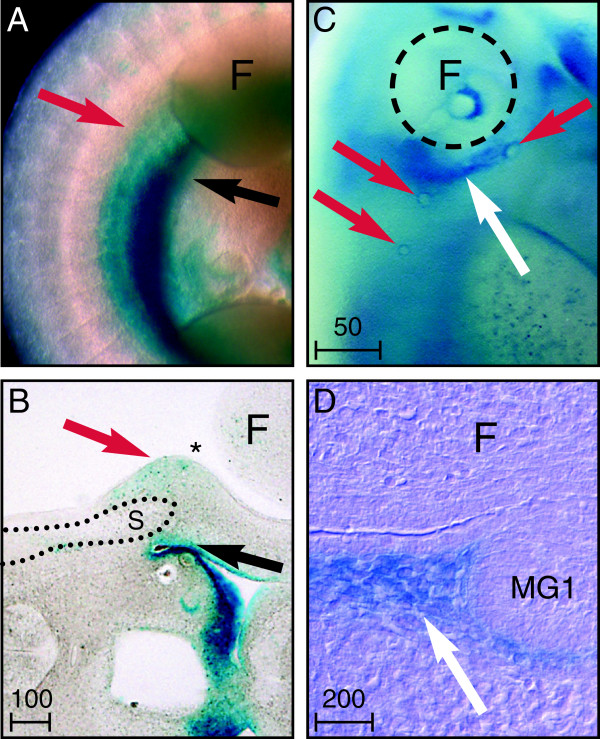
**Latent TGFβ binding protein (Ltbp)1L-LacZ appears early in embryonic mammary development.** The 5-bromo-4-chloro-3-indolyl-β-D-galactoside (X-Gal)-stained embryonic day (E) 10.5 embryo **(A)** and vibratome section **(B)** show a broad stripe of Ltbp1L-LacZ (blue stain) that localizes to viscera (black arrow). Faint staining (red arrow) is seen at the dorsal border of the mammary line (asterisk) overlying the somitic tips (S). **(C)** X-Gal-stained E12.0 to E12.5 embryos show reporter expression in a streak along the forelimb axilla (white arrow) and surrounding placodes 1 to 3 (red arrows). **(D)** Nuclear fast red-counterstained section of the same region shows a gradient of Ltbp1L-LacZ in the mesenchyme (white arrow) oriented towards mammary gland 1 (MG1). F = forelimb. Scale bars represent distance in microns.

**Figure 3:**
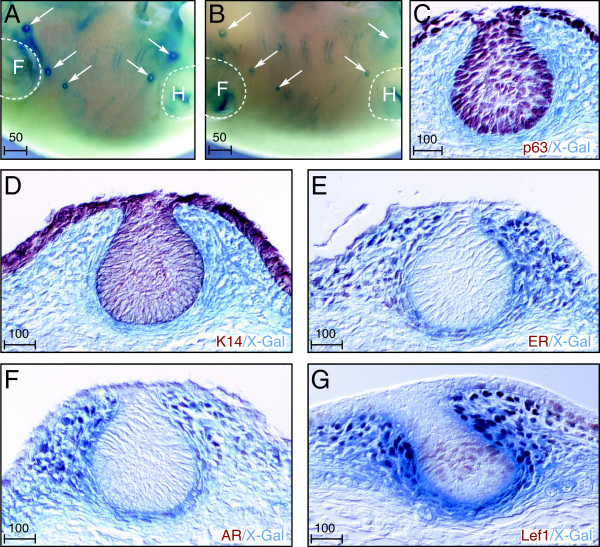
**Latent TGFβ binding protein (Ltbp)1L-LacZ is an early marker of mammary mesenchyme. (A)** The 5-bromo-4-chloro-3-indolyl-β-D-galactoside (X-Gal)-stained whole mount of embryonic day 14.5 female embryos. Ltbp1L-LacZ is expressed in a halo surrounding all mammary buds (white arrows). Expression is present but at lower levels in male embryos of the same stage (**B**, white arrows), coincident with the onset of sexual dimorphism. Localization of Ltbp1L-LacZ expression (blue stain) with immunohistochemical detection (brown stain) of epithelial markers p63 **(C)** and K14 **(D)**, as well as mammary mesenchymal markers estrogen receptor **(E)**, androgen receptor **(F)**, and lymphoid enhancer-binding factor 1 (Lef1) **(G)**. H = hindlimb, F = forelimb. Scale bars represent distance in microns.

We utilized an Ltbp1L^lz/+^ reporter mouse (Figure 1) in an attempt to understand potential physiological roles of LTBP1L [20]. Mammary development begins in mice at E10.5 with the formation of ectodermal ridges between the limbs, termed mammary lines that fragment to form placodes 3 and 4 [46]. Although Ltbp1L-LacZ expression was found between the limbs at this stage in *Ltbp1L*^*lz*/*+*^ embryos (Figure 2A black arrow), in sections it localized principally to internal viscera (Figure 2B black arrow). Robust Ltbp1L-LacZ expression first appeared at E11.5 to E12.0 in a subaxillary mesenchymal streak (Figure 2C and D, white arrow) abutting mammary placodes 1 and 2 (Figure 2C, red arrows). Intriguingly, ectodermal cells have been shown to migrate along a similar path to form pectoral and thoracic placodes 1 to 3 [47]. Later Ltbp1L-LacZ became intensely expressed around all five buds (Figure 3A-C) and colocalized with well-characterized mammary mesenchyme markers, such as androgen receptor (AR), estrogen receptor (ER), tenascin C and lymphoid enhancer-binding factor 1 (Lef1) (Figure 3D-G) [46]. Thus, during early embryonic mammary development *Ltbp1L* expression underlies a migratory route for epithelial cells and is one of the earliest markers of the inductive mammary mesenchyme.

### Mesenchymal *Ltbp1L* activity accompanies nipple induction and persists in smooth-muscle cells and myofibroblasts in the adult

**Figure 4:**
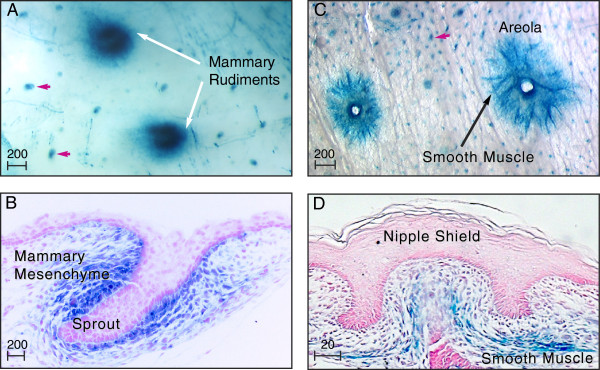
**Mesenchymal *Ltbp1L *activity is upregulated coincident with embryonic nipple formation. (A)** The 5-bromo-4-chloro-3-indolyl-β-D-galactoside (X-Gal)-stained skin whole mount (embryonic day16.5) shows robust Ltbp1L-LacZ expression (blue stain) in the mammary rudiments (white arrows) and developing hair follicles (red arrowheads). **(B)** Nuclear fast red (NFR)-counterstained section of the E16.5 mammary sprout shows strong focal Ltbp1L-LacZ expression in mammary mesenchyme at the onset of nipple induction. **(C, D)** Postnatal day 1 X-Gal-stained skin whole mount **(C)** and NFR-stained section **(D)** show Ltbp1L-LacZ expression in smooth-muscle cells at the base of the nipple shield extending radially into the developing areola. Scale bars represent microns.

In males, stimulation of androgen receptors at E14.5 induces mammary mesenchymal constriction and atrophy leading to bud loss and failure of nipple formation [48]. In contrast, in females, mammary mesenchyme signaling induces ductal morphogenesis, differentiation of nipple epithelium and suppression of hair follicles within the areola [49]. Reflecting this sexual dimorphism, Ltbp1L-LacZ expression was diminished in E14.5 males (Figure 3B) and lost by E15.5 but was maintained in females (Figure 3A), and robustly expressed during nipple induction at E16.5 (Figure 4A, B). Once the nipple shield had formed, reporter expression became restricted to muscle cells of the areola (Figure 4C, D).

**Figure 5:**
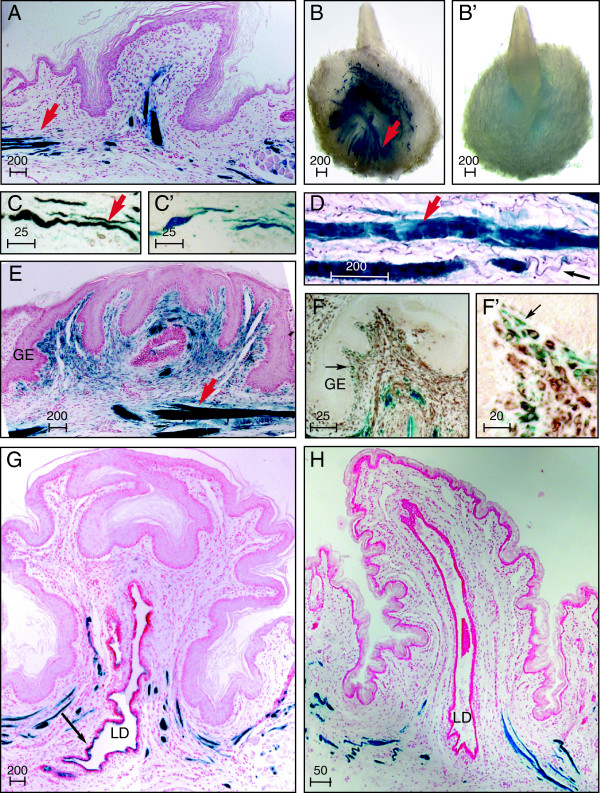
**Sustained latent TGFβ binding protein (Ltbp)1L-LacZ expression in the specialized stroma of the adult nipple. (A-E)** Robust Ltbp1L-LacZ expression was detected throughout postnatal development by 5-bromo-4-chloro-3-indolyl-β-D-galactoside (X-Gal) staining (blue stain) in bands of smooth-muscle cells (red arrows) at the base of the nipple underlying the areola in the region of the nipple sphincter **(A)** Histological section through a nipple of a virgin mouse, counterstained with nuclear fast red (NFR). **(B, B’)** Nipples from a pregnant (P14.5) mouse are shown from below **(B)** and above **(B’)**. **(C)** Immunohistochemical detection of SMA 1 (brown stain) colocalizes with Ltbp1L-LacZ in areola smooth muscle, P13.5. Secondary antibody control **(C’)**. **(D)** Resorcin-fuchsin stain detects elastin fibrils (black arrow to deep purple stain) encompassing Ltbp1L-LacZ-expressing smooth muscle. **(E)** NFR-stained section shows Ltbp1L-LacZ upregulation in nipple stromal cells during pregnancy, P13.5. **(F, F’)** Colocalization of Ltbp1L-LacZ expression (black arrow to blue stain) with immunohistochemical detection of vimentin (brown stain) in stromal cells underlying the nipple germinative epithelium (GE) just prior to clefting, during pregnancy (P15.5). NFR-counterstained nipple sections from mice during **(G)** pregnancy P16.5 and **(H)** involution. Note the absence of X-Gal staining in the region of the lactiferous duct (LD) within the nipple in **G** and **H** and acquisition of reporter expression as the LD enters the fat pad at the base of the nipple (see black arrow in **G**). Scale bars represent distance in microns.

Nipples undergo significant postnatal connective tissue remodeling. In virgin and early pregnant mice, the nipple, delimited by germinative epidermal ingrowths, encloses predominantly collagenous connective tissue. During late pregnancy, nipple stromal cells proliferate and synthesize elastin, leading to elastic fiber hypertrophy [50]. Ltbp1L-LacZ was strongly expressed in smooth muscle of the nipple sphincter, located at the base of the areola (Figure 5A-C), which were surrounded by elastic fibers (Figure 5D). *Ltbp1L* was silent within the nipple stroma at most developmental stages (Figure 5A, G, H). However, robust Ltbp1L reporter expression appeared briefly during mid-pregnancy P13.5 within vimentin-positive stromal cells (Figure 5E, F) at the base of the lactiferous duct and directly adjacent to the clefting germinative epithelium. Thus the temporal-spatial expression of *Ltbp1L* appears coincident with the formation of the nipple sphincter and during elastin synthesis by stromal myofibroblasts.

### *Ltbp1L* promoter activity coincides with ductal lumena formation within the embryonic mammary tree

**Figure 6:**
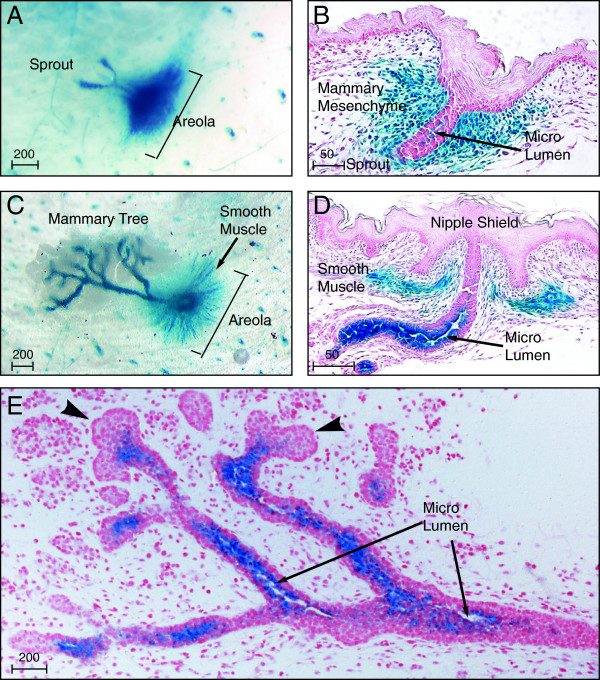
**Latent TGFβ binding protein (Ltbp)1L-LacZ appears at the onset of lumen formation. (A)** The 5-bromo-4-chloro-3-indolyl-β-D-galactoside (X-Gal)-stained whole mounts **(A, C)** and sections counterstained by nuclear fast red **(B, D, E)** at embryonic day (E) 17.5 **(A, B)**, postnatal day 1 P1 **(C, D)** and E18.5 **(E)**. Note Ltbp1L-LacZ appears as microlumen form within the mammary sprout (**B**, **D**, **E** arrows). Ltbp1L-LacZ is absent from the bulbous multilayered tips (**E**, arrowhead). Ltbp1L-LacZ is sustained in the mammary mesenchyme **(A, B)** and developing smooth muscle cells of the nipple areola **(C, D)**. Scale bars represent distance in microns.

At E16 mammary mesenchymal signaling induces proliferation of a solid cord of cells to form the mammary sprout [46,49]. *Ltbp1L* remained silent within the epithelium at this stage (Figure 4B) but became robustly expressed at around E17.5, in luminal cells coincident with the appearance of microlumen (Figure 6B, D). Intriguingly, reporter expression was absent from the multilayered ductal tips (arrowheads, Figure 6E) and from portions of the lactiferous duct within the nipple that comprise stratified epithelium (Figure 6D). Thus, *Ltbp1L* is induced only when the lactiferous duct enters the fat pad and differentiates into a bi-layered tube comprising a simple epithelial luminal lining surrounded by molecularly distinct basal cells.

### Ltbp1 mRNA is dynamically modulated during postnatal development

**Figure 7:**
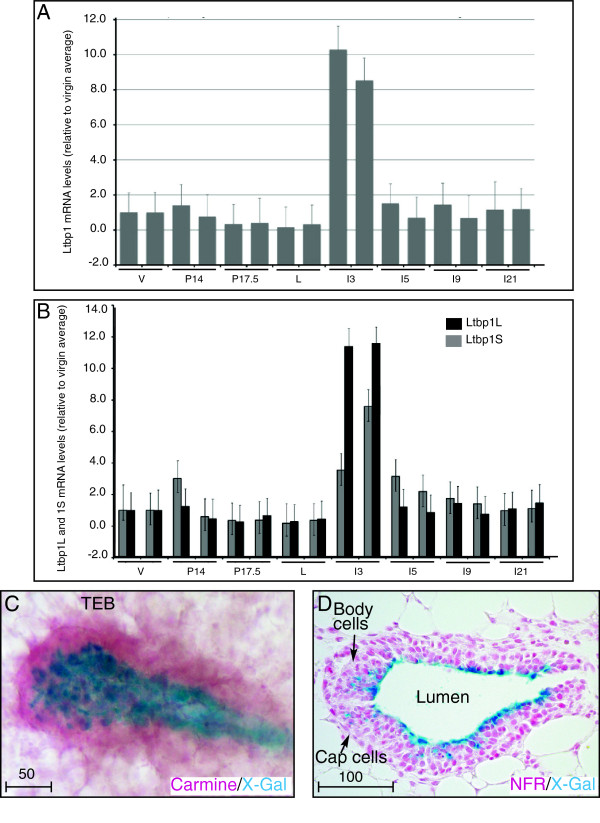
***Latent TGFβ binding protein *(Ltbp)*1L *expression in the postnatal mammary gland. (A)** Ltbp1 mRNA expression is highly elevated during early involution peaking 3 days after forced pup weaning. **(B)** Ltbp1L (black bars) and Ltbp1S (gray bars) are most abundant during early involution day 3. Total RNA from mammary gland tissues, harvested from various developmental time points (12-week virgin (V), 14 days pregnant (P14) and 17.5 days pregnant (P17.5), lactating (L), involution days 3 (I3), 5 (I5), 9 (I9) and 21 (I21)), was reverse transcribed and subjected to qRT-PCR. Ltbp1 **(A)** mRNA levels as well as those of Ltbp1L and Ltbp1S isoforms **(B)** were normalized to β2-microglobulin expression and plotted as levels relative to tissue from 12-week-old virgins. Error bars indicate standard deviation of the cycle threshold (Ct) values (n = 4). mRNA levels from two representative mouse samples for each time point are shown on the graphs. **(C, D)** Ltbp1L-LacZ (blue stain) expression in cells bordering the lumen of the terminal end bud (TEB). **(C)** In carmine-5-bromo-4-chloro-3-indolyl-β-D-galactoside (X-Gal)-stained whole mounts, Ltbp1L-LacZ localizes to the internal portion of TEB and is surrounded by non-expressing cap and body cell layers. Note the punctate appearance of X-Gal staining demonstrating that Ltbp1L-LacZ is in a subset of luminal cells. **(D)** Sections of the same 5-week-old virgin stained with nuclear fast red shows Ltbp1L-LacZ expression in cells bordering the lumen. Scale bars represent distance in microns.

Mammary development continues postnatally during puberty and is completed only after a first pregnancy. To determine if *Ltbp1* was expressed during the postnatal period we isolated total RNA from mammary glands of virgin, pregnant and lactating mice as well as from those undergoing post-parous remodeling (involution), and performed qRT-PCR. Total Ltbp1 mRNA was expressed at modest levels in virgin mice, decreased during pregnancy, lost during lactation and robustly upregulated during early involution, peaking at day 3 and returning to that found in virgins after 5 to 7 days (Figure 7A). Ltbp1S and 1 L showed a similar trend, however Ltbp1L rose in a more pronounced fashion at day 3 (Figure 7B).

### *Ltbp1L* is induced in ductal luminal cells and distinguishes them from alveolar lineages

To determine more precisely where the *Ltbp1L* promoter is activated during postnatal mammary development, we examined Ltbp1L-LacZ expression in whole mounts and histological sections. In pubertal mice a balance of proliferation and apoptosis within outer cap and inner multilayered body cells of the bulbous terminal end buds (TEBs) generates the permanent ductal tree and creates a lumen in the subtending ductal system. X-Gal-stained whole mounts revealed Ltbp1L-LacZ expression lining the lumen of the TEB (Figure 7C). Reporter expression was notably absent from the vast majority of body cells, which are considered to be actively proliferating luminal precursors (Figure 7D).

**Figure 8:**
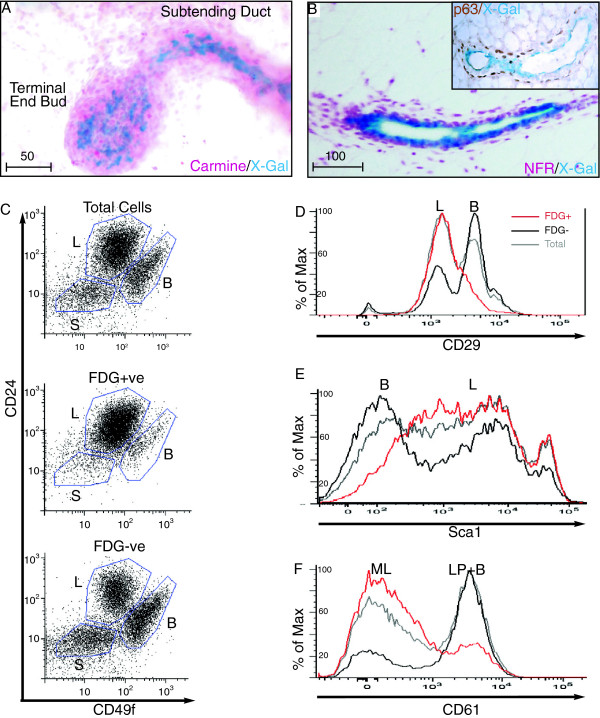
**A major subset of ductal luminal cells show *Ltbp1L *activity. (A)** Carmine/5-bromo-4-chloro-3-indolyl-β-D-galactoside (X-Gal)-stained whole mount of the terminal end bud, **(B)** nuclear fast red-stained section of the mature duct show Ltbp1L-LacZ expression in luminal cells, basal cells are visualized by immunohistochemcal detection of p63 (inset). Flow cytometry gating for FDG-Gal^+^ (Ltbp1L-LacZ)^+^ cells enriches for the luminal CD24^+^CD49^LO^ compartment (**C**, top panel). Ltbp1L-LacZ marks 65% of total luminal cells (**C**, middle panel), and 35% of luminal cells are Ltbp1L-LacZ-negative (**C**, bottom panel). Gates represent stromal *S*, luminal *L*, and basal *B* populations from two mature virgin mice. **(D-F)** Histograms show Ltbp1L-LacZ -positive cells (red) are CD29^LO^**(D)**, Sca-1^HI^**(E)** and CD61^LO^**(F)**. Data represents n = 2 replicates for all experiments. Peaks represent luminal *L*, basal *B*, mature luminal *ML* and luminal progenitors *LP*. Scale bars represent distance in microns.

Ltbp1L-LacZ was expressed prominently in luminal cells of the permanent ductal system (Figure 8A, B). To further characterize *Ltbp1L* activity within the luminal lineage we utilized a fluorescent β-Galactosidase substrate, FDG-Gal to detect Ltbp1L-positive cells by flow cytometry. Mammary stromal, basal, and luminal subpopulations can be separated by their differential expression of CD24, CD49f and CD29 (Figure 8C top panel) [51]: 65% of CD24^high^CD49f^low^ and CD29^low^ luminal cells (Figure 8C middle panel and 8D respectively) were FDG^+^ and therefore expressed Ltbp1L-LacZ. Interestingly, 35% of the luminal cell population was negative (Figure 8C bottom panel), consistent with our observation of a punctate X-Gal staining pattern in some whole mounts (Figure 8A). Luminal cells can be further defined into mature and progenitor populations by their expression of CD61, a marker of integrin β3 that is highly expressed in luminal progenitors and Sca1 [51]. The majority of FDG^+^ cells were Sca1^+^ (Figure 8E) and CD61^-^ (Figure 8F), but a small percentage was CD61^+^. Collectively these data indicate that *Ltbp1L* is induced in a subset of luminal progenitors and mature luminal cells of the permanent ductal system.

**Figure 9:**
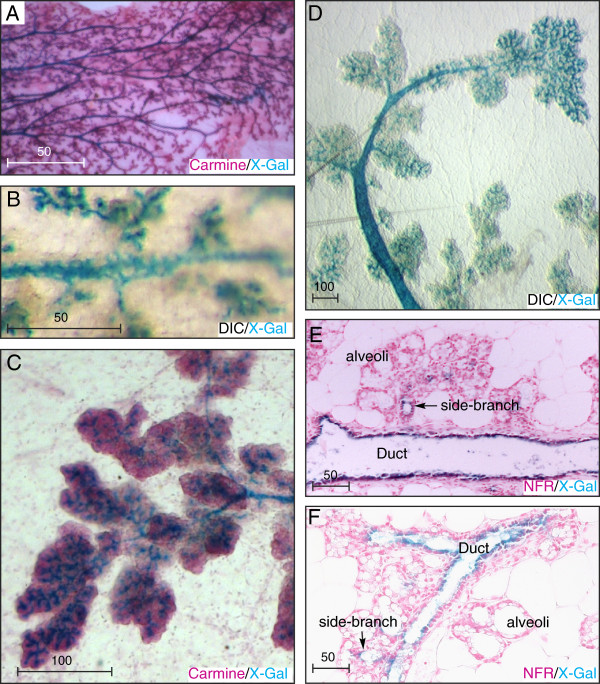
***Latent TGFβ binding protein *(*Ltbp*)*1L *is silent in secretory alveoli during pregnancy. (A-D)** The 5-bromo-4-chloro-3-indolyl-β-D-galactoside (X-Gal)-stained whole mounts show Ltbp1L-LacZ (blue stain) is expressed in ducts and side branches at all stages of pregnancy, but absent from alveoli. **(A)** Carmine/X-Gal-stained whole mount P13.5. **(B)** Ltbp1L-LacZ is expressed in a reticular staining pattern demarcating a subset of ductal cells. **(C)** Carmine/X-Gal-stained whole mount P14.5 **(D)** X-Gal-stained whole mount P14.5. **(E, F)** nuclear fast red(NFR)/X-Gal-stained sections P16.5 show Ltbp1L-LacZ is expressed in ducts but not in adjacent alveoli. Scale bars represent distance in microns.

Pregnancy initially induces extensive arborization of the mammary ductal tree. Alveolar clusters form on the tip of each side-branch during mid-pregnancy and undergo secretory differentiation during late pregnancy in preparation for lactation. Mammary whole mounts taken during early, mid and late pregnancy showed Ltbp1L-LacZ expression throughout the permanent ductal system and within the newly developing transient side branches (Figure 9A-D). In striking contrast, *Ltbp1L* remained silent within developing and differentiating alveoli throughout pregnancy (Figure 9C, D). Histological sections through p16.5 alveolar clusters confirmed that Ltbp1L-LacZ expression was restricted to ducts and side branches (Figure 9E, F) and absent from alveolar milk-producing cells that contained large lipid droplets (Figure 9F). Thus *Ltbp1L* is a rare and highly specific marker distinguishing ductal from alveolar luminal lineages.

### *Ltbp1L* activity is dramatically upregulated during involution

**Figure 10:**
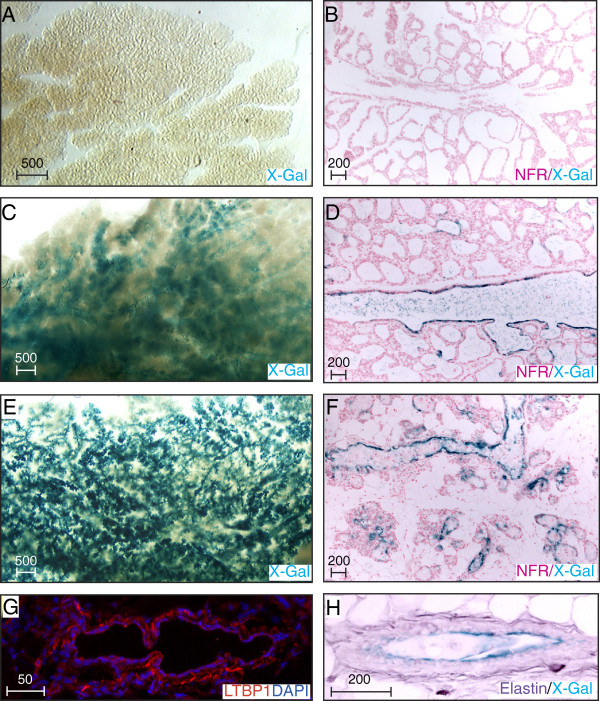
**Modulation of latent TGFβ binding protein (Ltbp)1L-LacZ expression during involution. (A, C, E)** The 5-bromo-4-chloro-3-indolyl-β-D-galactoside (X-Gal)-stained whole mounts and **(B, D, F)** nuclear fast red (NFR)-stained sections of lactating **(A, B)**, and involuting glands removed 24 h **(C, D)** and 72 h **(E, F)** after forced pup weaning. **(G)** Immunofluorescent detection of frozen mammary glands isolated from mice at involution day 5 using anti-LTBP1 (red) and counterstained with 4',6-diamidino-2-phenylindole (DAPI) (blue) to detect nuclei. **(H)** Elastic fibers (purple), identified by resorcin-fuchsin staining, on X-Gal-stained paraffin-embedded sections of mammary gland harvested from mice at involution day 21 shows periductal arrangement of fibrils encasing the permanent duct. Scale bars represent distance in microns.

During lactation luminal cells of both ducts and alveoli undergo secretory differentiation. Ltbp1L-LacZ expression was undetectable in whole mounts and sections at this stage (Figure 10A, B) consistent with the relative diminishment of Ltbp1 mRNA expression at this stage in qRT-PCR analysis (Figure 7A, B). Milk stasis and ductal distension trigger an initial phase of involution involving cell death that is reversible if suckling resumes [37,52]. After 48 h, however, involution proceeds irreversibly with collapse and removal of transient alveolar and side-branch structures. Throughout this process the permanent ductal system and resident stem cells are protected from destruction. Within 24 hours of pup weaning Ltbp1L-LacZ expression appeared along the main ducts and distended primary side branches (Figure 10C, D). In sections, the reporter expression appeared in a discontinuous pattern within a subset of luminal cells (Figure 10D) and was absent from alveoli, which remained morphologically distended. By 72 h, as the alveoli collapsed and were undergoing clearance, Ltbp1L-LacZ became robustly expressed within remaining luminal epithelia (Figure 10E, F). This sharp increase in LTBP1 expression around the transition to irreversible involution was confirmed by qRT-PCR where Ltbp1, 1 L and 1S mRNA peaked at 72 h (Figure 7A, B). Collectively these results show that both forms of *Ltbp1* are transcriptionally regulated throughout mammary development in a highly dynamic temporal and spatial manner and are maximal during involution.

### Ltbp1 and elastin encase the mammary ductal system

**Figure 11:**
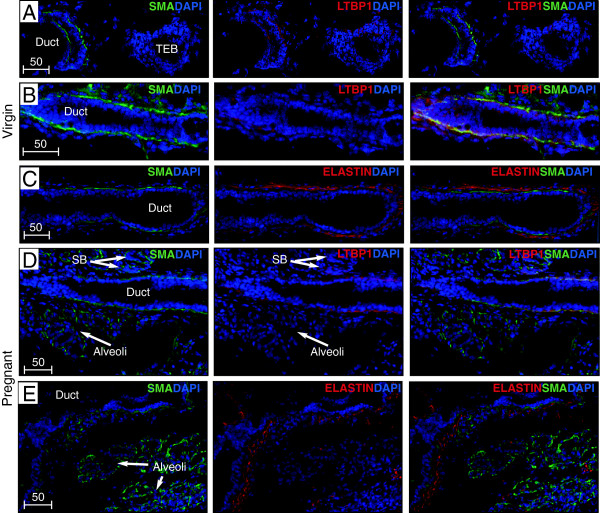
**Localization of latent TGFβ binding protein (Ltbp)1 and elastin in the pubertal and pregnant mammary gland.** Immunofluorescent detection of Ltbp1 **(A**, **B** and **D)**, elastin **(C*, *E)** and smooth-muscle actin (SMA) **(A-E)** in frozen sections of mammary glands isolated from 6-week-old pubertal **(A-C)** and 14.5-day pregnant mice **(D, E)**. Anti-SMA detected by Alexa-488 secondary antibodies (green) localized basal myoepithelial cells of mature ducts but not cap cells of the terminal end bud (TEB) and robustly stained basal cells of alveoli. Anti-Ltbp1 detected by Cy3-coupled secondary antibodies (red in center panels) in virgin **(A, B)** and pregnant glands **(D)** detected Ltbp1 deposited in close apposition to the SMA-positive basal cell layer (yellow in right merged panel). In contrast the cap cell layer that surrounds the TEB was negative with both anti-SMA and anti-Ltbp1 antibodies. Anti-tropoelastin detected by Cy3 coupled secondary antibodies (red in center and right panels) in virgin **(C)** and pregnant **(E)** glands detects elastic fibers surrounding the basal layer. Both Ltbp1 and elastic fibers are excluded from alveolar clusters. Frozen sections were viewed under a Carl Zeiss Confocal LSM510 microscope at 20× magnification and images were acquired using Zen Software version 2010. Scale bars represent distance in microns. DAPI, 4',6-diamidino-2-phenylindole.

Having identified the spatial pattern of *Ltbp1L* promoter activity and expression levels of both Ltbp1 mRNAs we next sought to determine the localization of the secreted Ltbp1L protein. We first examined sections of involuting glands by immunofluorescence and found Ltbp1L localized in microfibrillar strands surrounding ducts (Figure 10G). Resorcin-fuchsin detected elastic fibers in a similar periductal organization (Figure 10H). We next examined Ltbp1L and elastin organization at earlier stages (Figure 11). In pubertal glands SMA antibodies detected the basal cell layer of ducts (Figure 11A, B) but was absent from the contiguous cap cell layer of TEBs. Ltbp1 antibodies showed extensive regions of colocalization with SMA-positive basal cells along ducts but was also absent from the SMA-negative cap cell layer of TEBs indicating that Ltbp1 is deposited in close apposition to differentiated myoepithelial cells (Figure 11A-B). Weak Ltbp1 staining was seen in a few body cells of the TEB. The ductal system was also encased by a thick mesh of elastic fibers detected by anti-tropoelastin (Figure 11C). The elastic fibers, however, localized more distantly from the basal cell layer than Ltbp1. In glands from pregnant mice, Ltbp1 surrounded both the permanent ductal system and temporary side branches but was absent from alveolar clusters (Figure 11D). In contrast elastic fibers were restricted to the permanent ductal system (Figure 11E).

## Discussion

The importance of TGFβ signaling for mammary physiology and pathology has been well documented however the factors that regulate TGFβ presentation and activation are less well-understood [53]. Although LTBPs determine the spatial deposition of latent TGFβ and thus define the coordinates for its subsequent activation, surprisingly nothing is known about them in normal mammary gland. Here we show that *Ltbp1* is dynamically and focally regulated throughout mammary development. The major findings of our study are that 1) Within the mammary epithelium, *Ltbp1L* is transcribed exclusively by ductal luminal cells and distinguishes them from the alveolar luminal lineage; 2) Ltbp1 protein and elastic fibers exclusively encase the ductal system; 3) *Ltbp1L* and *1S* are upregulated during involution, a developmental window linked to high risk for breast cancer promotion; and 4) *Ltbp1L* is induced in mammary mesenchyme and sustained in the smooth-muscle cells of the nipple sphincter.

### *Ltbp1L* is induced in embryonic mammary mesenchyme and persists in nipple sphincter cells

Ltbp1L-LacZ is first expressed in an arc around the forelimb. This pattern is intriguing in light of reports that mammary precursors destined for placodes 1 to 3 migrate along a similar path [47]. It is well known that TGFβ signaling promotes EMT and motogenesis. Thus, *Ltbp1L* expression may designate a migratory route and potentially stimulate ectodermal cell migration by presenting a focal source of TGFβ. *Ltbp1L* is next upregulated in the specialized mammary mesenchyme, which plays a pivotal role in inducing mammary morphogenesis and specifying the embryonic nipple and areola [49]. To date there have been no reports of TGFβ involvement in these inductive processes, although other members of the TGFβ family, such as bone morphogenic protein (BMP)4, are known to play critical roles [54,55]. We find that the expression of mammary mesenchymal markers remains unperturbed and embryonic mammary development proceeds normally in *Ltbp1L*^*lz/lz*^ embryos, indicating that *Ltbp1L* is not essential for mammary mesenchyme specification or inductive function. These results do not, however, preclude the possibility that the products of *Ltbp1S*, which is expressed from an independent promoter, or other *Ltbp* genes may compensate [56]. Alternatively Ltbp1L may function at later stages in the differentiation of these cell types. Ltbp1L-LacZ expression persists within smooth muscle cells aligned in radial arrays under the areola, which facilitate nipple projection and regulate the nipple sphincter during milk let-down. There have been no studies on TGFβ in the nipple, however, misexpression of Wnt5a, a target gene of TGFβ, has been shown to impair milk ejection, supporting the concept that specific levels of TGFβ signaling may be critical for nipple function [57]. We also observe strong Ltbp1L-LacZ expression in myofibroblasts during mid-pregnancy when the stroma synthesizes elastin to provide structural support for the lactiferous duct [58]. Whether Ltbp1L functions to reinforce the surrounding elastic fibers, and/or serves in a mechanosensory capacity between TGFβ signaling and the establishment of the unique nipple stroma, remains to be determined.

### Ltbp1 and ductal cell fate

*Ltbp1L* activity is a consistent marker of the ductal luminal lineage, appearing in the embryo at the first sign of ductal canalization. This specificity is maintained throughout pubertal development and pregnancy where it serves as a rare marker distinguishing ductal from alveolar luminal cells. Transplantation studies have suggested that ductal and alveolar progenitors are distinct, but little is known about differences between mature ductal and alveolar luminal cell-types [59]. *Ltbp1L* is active in approximately 65% of luminal cells but silent within the inner body cells of the TEB, which are thought to be a proliferative progenitor population. It is upregulated within mature CD61^-^Sca1^+^ cells in the subtending duct and within a small subpopulation of CD61^+^ luminal progenitors, which we speculate may generate side branches during pregnancy. Previous studies have implicated TGFβ signaling in suppressing proliferation of luminal populations and maintaining the potency of basal stem cell populations [34,36,60]. Our results show that Ltbp1 protein is deposited in close apposition to basal cells encasing the ductal system and thereby positioning TGFβ to carry out these functions.

### Ltbp1 in the physiology of ductal dilation and distension

The appearance of Ltbp1L-LacZ expression coincident with lumen formation in the embryonic mammary rudiment and in the pubertal TEB suggests Ltbp1 may position TGFβ to generate lumen by inducing apoptosis [61]. TGFβ is a well-known pro-apoptotic cytokine and multiple studies have demonstrated a role for apoptotic factors in lumen formation *in vitro* and *in vivo*[34,37]. However the periductal restriction of Ltbp1 protein in close association with elastic fibers makes this function unlikely and moreover indicates that they participate in some ductal versus alveolar specific process. A distinguishing feature of ducts is that their lumen remain open at all times. Whether Ltbp1 serves to physically support the open ducts by reinforcing their elastic fiber encasement and/or positions TGFβ to monitor ductal lumenal diameter in a mechanosensory fashion remains to be determined.

### *Ltbp1L* is silenced during lactation and dramatically induced during involution

The most dramatic changes in *Ltbp1* activity occurred with the onset and cessation of lactation. Ltbp1, 1L and 1S mRNA were undetectable during lactation, and Ltbp1L-LacZ expression was lost even from the ducts as the entire epithelium assumed a secretory phenotype and the lumen became engorged with milk. This loss of *Ltbp1L* and 1S expression coincides with a change in the trafficking of latent TGFβ from basolateral secretion as a large latent complex destined for incorporation into the ECM in an Ltbp-dependent manner to apical secretion of small latent complex into milk, which functions to promote IgA production and induce oral tolerance in the newborn [62].

*Ltbp1L* is dramatically induced during involution. Involution is a biphasic event, marked by distinct biological processes. For up to 48 h after weaning the process is reversible and characterized by alveolar apoptosis. After this point it becomes irreversible, as protease-mediated matrix remodeling leads to alveolar collapse and rebuilding of the ECM, to return the gland to a virgin-like state [63]. Teat-sealing experiments have shown that ductal distension triggers involution even in the presence of circulating lactogenic hormones, highlighting the role of local factors [37]. Our results show that *Ltbp1L and 1S* are induced within 24 h and peak at day 3 of involution, remaining elevated for some days. This pattern is similar to that reported for TGFβ3 in several microarray studies [64,65]. TGFβ3 is upregulated 6-fold within 3 h of weaning and has been implicated as a local factor triggering alveolar apoptosis, however, the mechanism for its activation has not been studied [37]. Whether *Ltbp1* is expressed early enough to facilitate TGFβ3’s role in apoptosis remains to be determined. The peak of *Ltbp1* and TGFβ3 induction correlates with the transition to the irreversible stage of involution, suggesting that elevated TGFβ signaling may contribute to this transition. Little is known about the role of TGFβ3 in later involution, though it has been hypothesized to promote fibroblast migration and ECM generation based on the upregulation of wound healing and ECM genes that are targets of TGFβ signaling during this phase [17,63,65]. Alternatively, the localization of Ltbp1 protein along ducts suggests it may function to protect the permanent ductal system and its ductal stem cells from destruction by integrating integrin and TGFβ signaling, which promote cell survival and stem cell potency, respectively [66]. Lastly, our finding that *Ltbp1* expression is dramatically elevated during involution, when taken collectively with the fact that *LTBP1* appears in two metastatic signatures [5,6] and regulates TGFβ, a factor inducing EMT, suggests that LTBP1 may be a prometastatic element in pregnancy-associated breast cancer (PABC). Detected postpartum, PABC is highly aggressive and this feature is thought to result from the action of prometastatic factors in the microenvironment of involuting glands [67]. Thus LTBP1 levels may be worthy of investigation as a risk factor.

## Conclusions

In conclusion, our results establish that *Ltbp1* is dynamically regulated during mammary development. The pattern of *Ltbp1L* activity and Ltbp1 protein localization suggest roles in reinforcing elastic support and mechanosensory feedback for mammary ducts and nipple. Currently nothing is known of the role of this important TGFβ regulator in human breast. Its elevation during involution suggests LTBP1 is worthy of further investigation as a prometastatic candidate in PABC.

## Abbreviations

AR: Androgen receptor; BAC: Bacterial artificial chromosome; BMP: Bone morphogenetic protein; Bp: Base pairs; CD: Cluster of differentiation; DAPI: 4',6-diamidino-2-phenylindole; ECM: Extracellular matrix; EDTA: Ethylenediaminetetraacetic acid; EGF: Epidermal growth factor; EMT: Epithelial to mesenchymal transition; ER: Estrogen receptor; ES: Embryonic stem; FAM: 6-carboxyfluorescein (FAM); Fb: Fibrillin; FDG: Fluorescein Di-β-D-Galactopyranoside; FN: Fibronectin; HRP: Horseradish peroxidase; IHC: Immunohistochemistry; LAP: Latency-associated propeptide; Lef1: Lymphoid enhancer-binding factor 1; LLC: Large latent complex; LTBP: Latent TGFβ binding protein; MGB: Dihydrocyclopyrroloindole tripeptide minor groove binder; NFR: Nuclear fast red; PABC: Pregnancy-associated breast cancer; PBS: Phosphate buffered saline; PFA: Paraformaldehyde; qRT-PCR: Quantitative reverse transcriptase-polymerase chain reaction; RER: Rough endoplasmic reticulum; RGD: Arginine-glycine-aspartic acid; Sca1: Stem cell antigen 1; SLC: Small latent complex; SMA: Smooth muscle actin; SP: Signal peptide; TEB: Terminal end bud; TGFβ: Transforming growth factor β; TGF-βR: Transforming growth factor β Receptor; X-Gal: 5-bromo-4-chloro-3-indolyl-β-D-galactoside.

## Competing interests

The authors declare that they have no competing interests.

## Authors’ contributions

AC, JS and AP performed all the experiments in the manuscript (including characterization of Ltbp1 promoter activity throughout mammary development, isolation of RNA from various stages of the postnatal mammary gland for qRT-PCR analysis, flow cytometric analysis of postnatal mammary gland to define ductal luminal populations within the postnatal mammary gland, characterization of Ltbp1 protein expression in virgin, pregnant and involuting mice by immunofluorescence and of elastic fibers by immunofluorescence and resorcin-fuchsin staining), analyzed the data and drafted the manuscript. MH performed the initial characterization of Ltbp1L-LacZ expression on whole-mount embryos at early embryonic stages (E10.5 to E14.5). GD carried out the qRT-PCR amplification of *Ltbp1* and its isoforms *Ltbp1L* and *Ltbp1S* and analyzed their expression levels in the postnatal mammary gland. DF engineered the original *Ltbp1L*^*lz/+*^ mouse by targeted insertional mutagenesis and analyzed the expression levels of *Ltbp1*, *Ltbp1L* and *Ltbp1S* in the postnatal mammary gland. PC conceived of the study, participated in the design of all experiments, coordinated and drafted the manuscript. All authors read and approved the final manuscript.
